# Complexity and specificity of the maize (*Zea mays* L.) root hair transcriptome

**DOI:** 10.1093/jxb/erx104

**Published:** 2017-04-08

**Authors:** Stefan Hey, Jutta Baldauf, Nina Opitz, Andrew Lithio, Asher Pasha, Nicholas Provart, Dan Nettleton, Frank Hochholdinger

**Affiliations:** 1 INRES, Institute of Crop Science and Resource Conservation, Crop Functional Genomics, University of Bonn, D-53113 Bonn, Germany; 2 Department of Statistics, Iowa State University, Ames, IA 50011-1210, USA; 3 Department of Cell and Systems Biology, University of Toronto, Toronto, ON, M5S 3B2, Canada

**Keywords:** eFP browser, maize, phylogeny, RNA sequencing (RNA-seq), root hair, single cell analysis, transcriptome.

## Abstract

Root hairs are tubular extensions of epidermis cells. Transcriptome profiling demonstrated that the single cell-type root hair transcriptome was less complex than the transcriptome of multiple cell-type primary roots without root hairs. In total, 831 genes were exclusively and 5585 genes were preferentially expressed in root hairs [false discovery rate (FDR) ≤1%]. Among those, the most significantly enriched Gene Ontology (GO) functional terms were related to energy metabolism, highlighting the high energy demand for the development and function of root hairs. Subsequently, the maize homologs for 138 Arabidopsis genes known to be involved in root hair development were identified and their phylogenetic relationship and expression in root hairs were determined. This study indicated that the genetic regulation of root hair development in Arabidopsis and maize is controlled by common genes, but also shows differences which need to be dissected in future genetic experiments. Finally, a maize root view of the eFP browser was implemented including the root hair transcriptome of the present study and several previously published maize root transcriptome data sets. The eFP browser provides color-coded expression levels for these root types and tissues for any gene of interest, thus providing a novel resource to study gene expression and function in maize roots.

## Highlight

This transcriptome study highlights genes exclusively and preferentially expressed in maize root hairs and their suggested functions, and provides a starting point for future genetic analyses.

## Introduction

Root hairs are tubular outgrowths of single epidermal cells instrumental for nutrient uptake and optimal plant development (Gilroy and Jones, 2000). The epidermis of plant roots comprises two types of cells: trichoblasts and atrichoblasts. While trichoblasts give rise to root hairs, atrichoblasts do not form root hairs. Three types of epidermal patterning have been observed in plant roots (reviewed in Dolan, 1996). In maize and some other monocots, most ferns, and most dicots, type I root hair patterning occurs, in which any root epidermis cell can randomly form a root hair. In type II root hair patterning, root hairs develop from the smaller cell of an asymmetric division. This type is implemented among early land plants, some monocot species, including rice, and the dicot family Nymphaeaceae. Type III root hair patterning occurs among some members of the Brassicaceae. The molecular mechanisms underlying the third type of root hair patterning have been extensively studied in the model species Arabidopsis (reviewed in Grebe, 2012; Hochholdinger and Nestler, 2012), in which only epidermis cells situated in a cleft between two underlying cortical cells (H-position) give rise to root hairs (Salazar-Henao *et al.*, 2016). In Arabidopsis, the positional cue is perceived by the leucine-rich repeat receptor-like kinase SCRAMBLED (SCM). The transcription factors WEREWOLF (WER), CAPRICE (CPC), and GLABRA2 (GL2) maintain the epidermal pattern in Arabidopsis under the control of SCM (Datta *et al.*, 2011). In contrast to Arabidopsis, the molecular mechanisms of other modes of root hair patterning remain largely elusive (Marzec *et al.*, 2015). Root hair development can be divided into three stages (Marzec *et al.*, 2015). At the first stage, cell specification into trichoblasts and atrichoblasts is determined. During the following initiation stage, a protrusion or bulge forms at the site of hair outgrowth. Finally, at the third stage, the root hair shaft elongates (Marzec *et al.*, 2015).

In Arabidopsis, once cell fate is determined, the basic helix–loop–helix (bHLH) transcription factors ROOT HAIR DEFECTIVE 6 (RHD6) and ROOT HAIR DEFECTIVE 6-LIKE 1 (RSL1) initiate root hair development (Menand *et al.*, 2007). Homologs of those genes have been shown to control root hair development in several other species including *Physcomitrella patens* (Jang *et al.*, 2011), *Brachypodium distachyon* (Kim and Dolan, 2016), rice (Kim *et al.*, 2017), and *Marchantia polymorpha* (Proust *et al.*, 2016). Further genes encoding bHLH transcription factors including *rsl2*, *rsl3*, *rsl4*, and *ljrhl1-like 3* (*lrl3*) are direct targets of RHD6 and RSL1 driving the development of root hairs (Yi *et al.*, 2010; Bruex *et al.*, 2012). Auxin has been demonstrated to trigger root hair development via the transcription factors RSL2 and RSL4 independently of RHD6/RSL1 (Yi *et al.*, 2010). Moreover, the duration of RSL2 and RSL4 expression is correlated with the final root hair length (Yi *et al.*, 2010; Datta *et al.*, 2015). Single mutants of the bHLH transcription factor encoding the genes *lrl1*, *lrl2*, and *lrl3* develop root hairs, while root hairs are absent in double and triple mutants of these genes, indicating partial genetic redundancy (Karas *et al.*, 2009). However, detailed histological analyses of root hairs in these mutants demonstrated that root hairs of the mutants *lrl1* and *lrl2* display an abnormal morphology and show branching which is not observed in wild-type plants, while *lrl3* mutants form shorter root hairs. Among these three genes, only *LRL3* expression is up-regulated upon auxin treatment (Bruex *et al.*, 2012). The *rsl2/rsl4* double mutant is unable to trigger root hair growth during auxin treatment, indicating that LRL3 alone is not sufficient for root hair growth (Yi *et al.*, 2010). Together, these transcription factors regulate the expression of root hair-specific genes (Bruex *et al.*, 2012) such as *CAN OF WORMS 1* (*COW1*) which encodes a phosphatidylinositol transfer protein (Grierson *et al.*, 1997), and *EXPANSIN A7* (*EXP7*) encoding a cell wall-loosening protein which acts during root hair initiation (Cho and Cosgrove, 2002).

In maize, six mutants affected in root hair elongation (*rth1*–*rth6*) have been identified. Four of the genes impaired in these mutants have been cloned thus far. The *rth1* gene encodes the SEC3 subunit of the exocyst complex which controls exocytotic growth of the root hair tip (Wen *et al.*, 2005). Moreover, *rth3* encodes a COBRA-like protein involved in secondary cell wall organization (Hochholdinger *et al.*, 2008). Furthermore *rth5* translates into an NADPH oxidase required for root hair elongation (Nestler *et al.*, 2014) while *rth6* gives rise to a cellulose synthase-like D cell wall protein (Li *et al.*, 2016).

Transcriptome and proteome studies are useful tools for advancing understanding of root hair function (Hossain *et al.*, 2015; Wang *et al.*, 2016). In Arabidopsis, a transcriptomic study identified a set of 208 ‘core’ root epidermal genes (Bruex *et al.*, 2012). Root hair transcriptomes after rhizobia infection were analyzed in *Medicago truncatula* (Breakspear *et al.*, 2014) and soybean (Libault *et al.*, 2010). For maize, the soluble proteome of root hairs obtained from 4-day-old primary roots was described (Nestler *et al.*, 2011).

The major goal of this study was to identify genes exclusively or preferentially expressed in root hairs of the maize inbred line B73 as a resource for future genetic analyses. In this context, we planned to establish a maize root view of the eFP browser to make these data easily accessible. Finally, we intended to identify maize homologs of genes known to be involved in Arabidopsis root hair development by phylogenetic reconstructions to study similarities and differences of these homologs in monocot and dicot model species.

## Materials and methods

### Plant material and growth conditions

Seedlings of the maize inbred line B73 were grown for 3 d in germination paper rolls (Anchor Paper, Saint Paul, MN, USA) as previously described, in constant darkness at 28 °C (Hetz *et al.*, 1996). Primary roots of 3–4 cm length were dipped in liquid nitrogen and root hairs were scraped off and collected as previously described (Li *et al.*, 2016). The remaining primary roots without root hairs were collected separately. Four biological replicates of root hairs and primary roots without root hairs were sampled, representing samples obtained from 150 primary roots per replicate.

### Auxin treatment

1-Naphthalic acetic acid (1-NAA; Sigma Aldrich, St. Louis, MO, USA) at 1 mg ml^–1^ was dissolved in 1 M KOH and diluted in deionized water to a final concentration of 5 µM. B73 seedlings were pre-germinated in wet paper rolls for 5 d in deionized water. A 5 ml aliquot of 1-NAA solution was pipetted directly onto each root. Seedlings were rolled in new paper rolls soaked in 1-NAA solution and incubated for 1 h or 3 h. Whole primary roots were harvested and shock frozen in liquid nitrogen immediately.

### Discovery of homologs and phylogenetic analyses

The iRootHair database (www.iroothair.org) lists a set of 138 Arabidopsis genes involved in root hair formation (Kwasniewski *et al.*, 2013) as of 1 June 2016. The maize homologs of these Arabidopsis genes which were identified based on amino acid sequence alignments according to the method of Li *et al.* (2003) are available at http://rice.plantbiology.msu.edu/ (Kawahara *et al.*, 2013). The list of maize genes was used as a starting point and corrected using a more precise prediction based on phylogeny as subsequently described: the predicted amino acid sequences were compared via tblastp searches against the translated nucleotide databases of *Clamydomonas reinhardii*, *Physcomitrella patens*, *Selaginella moellendorffii*, *Arabidopsis thaliana*, *Brachypodium distachyon*, *Medicago truncatula*, *Setaria italica*, *Gossypium raimondii*, *Solanum lycopersicum*, *Sorghum bicolor*, *Zea mays*, and *Oryza sativa* from the Phytozome 10.1 plant genomics portal (http://phytozome.jgi.doe.gov/pz/portal.html). All homologous sequences were downloaded and aligned using the MUSCLE algorithm in MEGA6 (Tamura *et al.*, 2013). For the generation of Bayesian trees, the alignments were exported into a NEXUS file and trees were generated by MrBayes (Huelsenbeck and Ronquist, 2001; Ronquist and Huelsenbeck, 2003). Trees were calculated using a Markov chain Monte Carlo approach with three hot chains and at least 5 million generations. Maximum likelihood trees were calculated with MEGA6 with 1000 bootstraps. Subsequently, phylogenetic trees were built using FigTree software (http://tree.bio.ed.ac.uk/software/figtree/). A total of 170 maize homologs were identified. Arabidopsis genes for which the phylogenetic relationship to maize genes has been adjusted compared with Kawahara *et al.* (2013) are marked with an asterisk in Supplementary Table S4 at *JXB* online. Groups of genes for which a one to one assignment was not possible were combined as indicated in Supplementary Table S4.

### RNA extraction and gene expression profiling

Plant tissue was ground in a pre-cooled mortar using liquid nitrogen. Total RNA was extracted from 60 mg of ground root tissue using the RNeasy Plant Mini Kit (Qiagen, Venlo, The Netherlands). RNA quality was assessed via an Agilent RNA 6000 Nano LabChip^®^ on an Agilent 2010 Bioanalyzer (Agilent Technologies, Santa Clara, CA, USA). RNA integrity number (RIN) values were between 7.9 and 9.8. The cDNA libraries for RNA sequencing were constructed using the TruSeq™ RNA Sample Prep Kit according to the protocol of the manufacturer (Illumina, San Diego, CA, USA). Samples were sequenced on an Illumina HiSeq™ 4000 instrument (Illumina). Trimming, mapping, and read count determination were performed with CLC Genomics Workbench (version 8.0.3; Qiagen). Only sequences for which 50% of the sequence matched the reference genome with at least 90% identity were considered for further analysis. Stacked reads with identical start and end co-ordinates were merged into one read. Reads were mapped to the filtered gene set of maize (FGS v2; Release 5b, ftp://ftp.gramene.org/pub/gramene/maizesequence.org/release-5b/filtered-set/) (Schnable *et al.*, 2009). Only reads mapping with at least 80% of their length with 90% sequence identity to unique positions of the genome were considered for further analyses. The sequencing data can be accessed through the NCBI Sequence Read Archive (SRA; http://www.ncbi.nlm.nih.gov/sra) accession no. SRP074164.

For quantitative real-time PCR (qRT-PCR) analyses, cDNA was synthesized from 1 µg of total RNA using the Quanta qScript™ cDNA SuperMix (Quanta, Gaithersburg, MD, USA). A 1:1 dilution series in factor two steps was prepared up to 1:128. Each biological replicate was measured in a BioRad CFX 384 Real-Time System (Biorad, Hercules, CA, USA) in three technical replicates using the Quanta PerfeCTa^®^ SYBR^®^ Green SuperMix (Quanta). Oligonucleotide primer efficiencies were calculated according to Bustin *et al.* (2009): PCR amplification efficiency=10^–1/slope^–1. Primer efficiencies were between 85% and 100%, and *R*^2^ was >0.95. Expression levels were calculated relative to a homolog of a myosin heavy chain gene (GenBank accession no. AI941656) previously used as a reference for expression in maize roots (Hoecker *et al.*, 2008) and to a putative ubiquitin carrier protein (GRMZM2G085600).

### Statistical analysis

#### Statistical procedures to determine gene activity

As in Tai *et al.* (2016), the transcriptional activity status of all genes (active/inactive) in root hairs and roots without hairs was determined using a generalized linear mixed model with a negative binomial response. The log of the mean was assumed to be a linear combination of fixed effects and random effects, plus sample- and gene-specific normalization factors (described below). Each sample type was represented by a fixed effect, and random effects were included to account for variation across biological replicates. The log of the TMM normalization factor (Robinson and Oshlack, 2010) was added to the linear predictor for normalization across samples, and a smooth function of gene length and GC content was used to normalize across genes.

The vector of fixed effects for each gene was assumed to be a draw from a multivariate normal distribution with an unknown and unrestricted mean and an unknown diagonal variance–covariance matrix. The precision of the random effects was assumed to follow a gamma distribution with unknown shape and rate. For each gene, the log of the negative binomial dispersion parameter was assumed to be constant and a draw from a normal distribution with unknown mean and variance. An empirical Bayes procedure via the R package ‘ShrinkBayes’ (Van De Wiel *et al.*, 2013) was used to estimate the unknown parameters, and to approximate the posterior distribution for the fixed effect associated with gene (g) and sample type (s) using the integrated nested Laplace approximation (Rue *et al.*, 2009).

The activity status of each gene was determined by computing Pgs (T), the posterior probability that the fixed effect for gene (g) sample type (s) was larger than a given threshold T. A gene (g) was called active for sample type (s) if Pgs (T) >0.5 and otherwise inactive. This method classifies genes as active or inactive based on the posterior distribution of fixed effects considering raw-read count, sequencing differences from sample to sample, gene length, and GC content differences, and therefore is more accurate than calls based only on a single raw read count threshold applied to all genes.

#### Analysis of differential gene expression

For the analysis of differential gene expression, only active genes which show an expression median greater than a threshold of 3.71 were included. The differentially expressed genes between the root hairs and the roots without root hairs were determined with the Bioconductor package limma (Smyth, 2005) in R (R version 3.1.1 2014-07-10, limma_3.20.9) as previously described in Baldauf *et al.* (2016). In brief, the raw sequencing reads were normalized by sequencing depth and log_2_-transformed to meet the assumptions of linear models. Furthermore, the mean–variance relationship within the count data was estimated and precision weights for each observation were computed (Law *et al.*, 2014). A linear model was fitted, consisting of a fixed effect for treatment, namely root hairs and roots without hairs, and block and a normally distributed random error term. Based on an empirical Bayes approach of the fitted data, hypotheses tests were performed using the contrasts.fit function of the Bioconductor package limma (Smyth, 2005). The resulting *P*-values were adjusted for multiple testing by controlling the false discovery rate (FDR) ≤1% (Benjamini and Hochberg, 1995).

### Maize root eFP browser

RPKM (reads per kilobase million) and FPKM (fragments per kilobase million) and values of various RNA sequencing (RNA-seq) root data sets from our laboratory (Opitz *et al.*, 2014, 2016; Tai *et al.*, 2016) were uploaded into the Maize eFP browser (Winter *et al.*, 2007) of the Bio-Analytic Resource (BAR) database. Representative images of maize roots at different developmental stages were created and an XML file was generated to power a new ‘root’ view within the Maize eFP Browser at http://bar.utoronto.ca/efp_maize/cgi-bin/efpWeb.cgi?dataSource=Maize_Root.

### GO term analysis

A Gene Ontology (GO) term analysis was conducted for genes preferentially expressed in root hairs (FDR ≤1%; 5585 genes) and genes preferentially expressed in roots without root hairs (FDR ≤1%; 14 708 genes) using singular enrichment analysis (SEA) with the online agriGO platform (bioinfo.cau.edu.cn/agriGO/; Du *et al.*, 2010). All expressed genes were used as reference.

## Results

### The maize root hair transcriptome

**Fig. 1.**
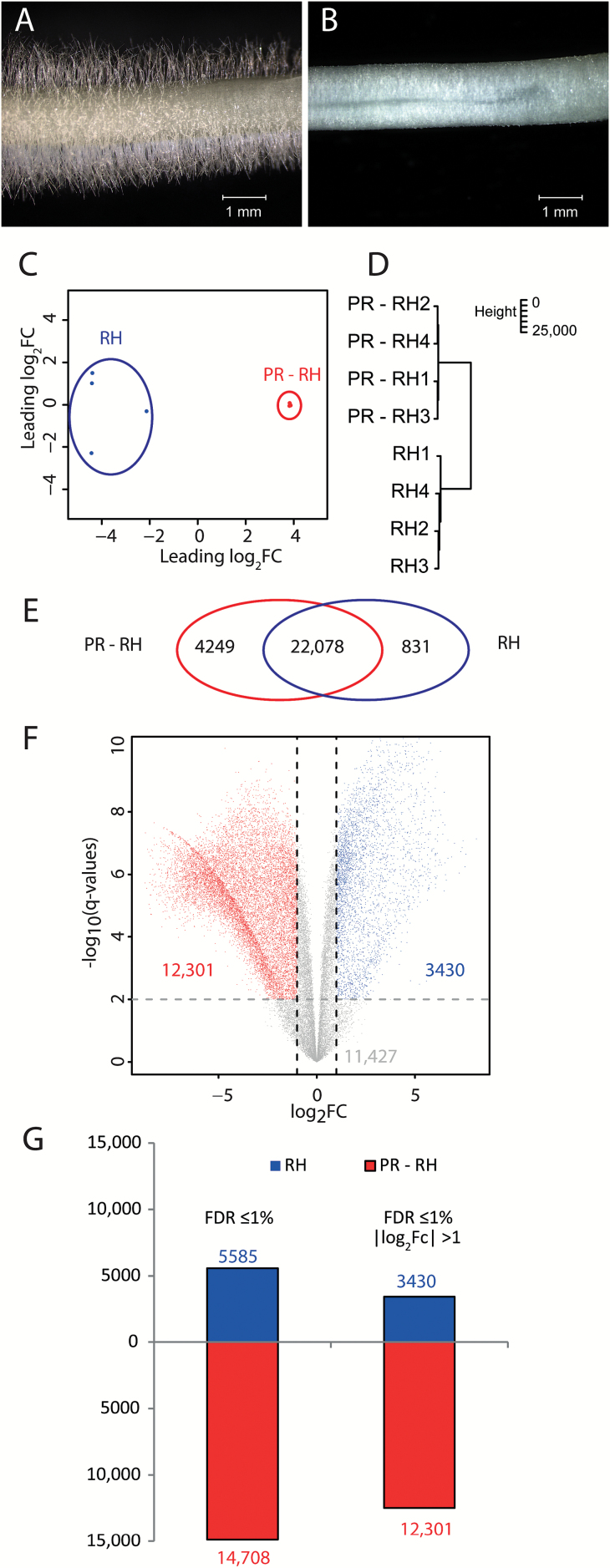
Sampling concept and RNA-seq results. (A) Primary roots with root hairs and (B) after root hairs were scraped off. (C) MDS plot and (D) hierarchical clustering of RNA-seq samples. Blue indicates root hair (RH) samples and red indicates primary roots without root hairs (PR – RH) samples. (E) Venn diagram of expressed genes. Red indicates genes expressed in PR – RH samples and blue indicates genes expressed in RH samples. (F) Volcano plot of expressed genes. Blue indicates genes preferentially expressed in RH and red indicates genes preferentially expressed in PR – RH (FDR ≤1%, |log_2_Fc| >1). (G) Numbers of differentially expressed genes (DEGs) in RH (blue) and PR – RH (red). Left bars represent DEGs (FDR ≤1%) and right bars represent DEGs (FDR ≤1%, |log_2_Fc| >1).

To generate a reference transcriptome map, maize root hairs were collected by dipping 3-day-old primary roots into liquid nitrogen and scraping off root hairs with a spatula. This procedure removed the large majority of root hairs from the primary roots (Fig. 1A, B). Total RNA was extracted from root hairs and from primary roots without root hairs. After enrichment, mRNA was transcribed into cDNA and subsequently sequenced on an Illumina HiSeq 4000 instrument. RNA-seq yielded on average 24.5 million 100 bp paired-end reads per sample (Supplementary Table S1). After quality trimming and removal of stacked reads, ~62% of remaining reads mapped uniquely to the maize filtered gene set (ZmB73_RefGen_v2, FGSv2, release 5b.60) that includes 39 656 high confidence gene models (Supplementary Table S1). A multidimensional scaling (MDS) plot revealed clustering of replicate samples for root hairs and for primary roots without root hairs, highlighting the reproducibility of the transcriptomics experiment (Fig. 1C). Large distances between the transcriptome samples of root hairs and primary roots without root hairs demonstrated their transcriptomic disparity (Fig. 1C). This finding was also substantiated by a hierarchical cluster analysis of the different samples (Fig. 1D).

To obtain a qualitative overview of global gene expression (i.e. active genes versus inactive genes), the activity status of each gene model of the filtered gene set was determined based on a generalized linear model in root hairs and in primary roots without root hairs (see the Materials and methods). In total, 27 158 active genes were identified (Fig. 1E; Supplementary Table S2). Among those, 831 (3%) were exclusively active in root hairs and 4249 (16%) in primary roots without root hairs. The majority of 22 078 (81%) genes were constitutively active in both tissues. Subsequently, among all genes declared ‘active’, differential gene expression was determined (FDR ≤1%; see the Materials and methods). Among differentially expressed genes, 14 708 genes were preferentially expressed in primary roots without root hairs while 5589 genes were preferentially expressed in root hairs. Most of the differentially expressed genes (12 301 in primary roots without root hairs and 3430 in root hairs) displayed an absolute |log_2_Fc| >1 (Fig. 1F, G). In general, genes preferentially expressed in root hairs showed a low average fold change (average Fc=1.7) compared with genes preferentially expressed in primary roots without root hairs (average Fc=3). These data indicate that root hairs representing a single cell type displayed less transcriptomic diversity than whole primary roots composed of multiple cell types.

### Over-represented biological processes among root hair genes

**Fig. 2.**
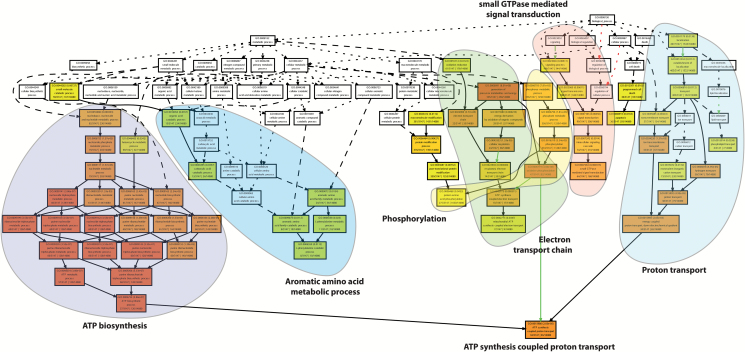
GO term enrichment. GO term analysis of genes preferentially expressed in root hairs. GO terms related to one pathway are highlighted by colored clouds. Colors of individual boxes indicate the significance level of enrichment. Significance levels are indicated in each box and range from yellow: *P*-value ≥0.05 to dark red: *P*-value ≥9.9e^–12^.

Genes preferentially expressed in root hairs (FDR ≤1% in comparison with primary roots without root hairs samples) were categorized according to GO functional terms. An enrichment analysis revealed in total 115 over-represented GO terms (Fig. 2; Supplementary Table S3). Many enriched terms were closely related to or were subcategories of each other. The most significantly enriched GO terms were related to energy metabolism, including ATP biosynthesis, phosphorylation, electron transport chain, and proton transport. Further GO categories related to small GTPase-mediated signal transduction and aromatic amino acid metabolic process were enriched (Fig. 2; Supplementary Table S3). Together, these data highlight the high activity of genes related to energy metabolism, suggesting a higher energy demand for the growth and function of root hairs than the remaining parts of the root. The most significantly enriched GO terms in primary roots without root hairs were related to translation and nucleosome assembly. Furthermore, GO terms related to hormone stimulus response were enriched (Supplementary Fig. S1).

### Implementation of the maize root hair and other maize root transcriptomes into the eFP browser

**Fig. 3.**
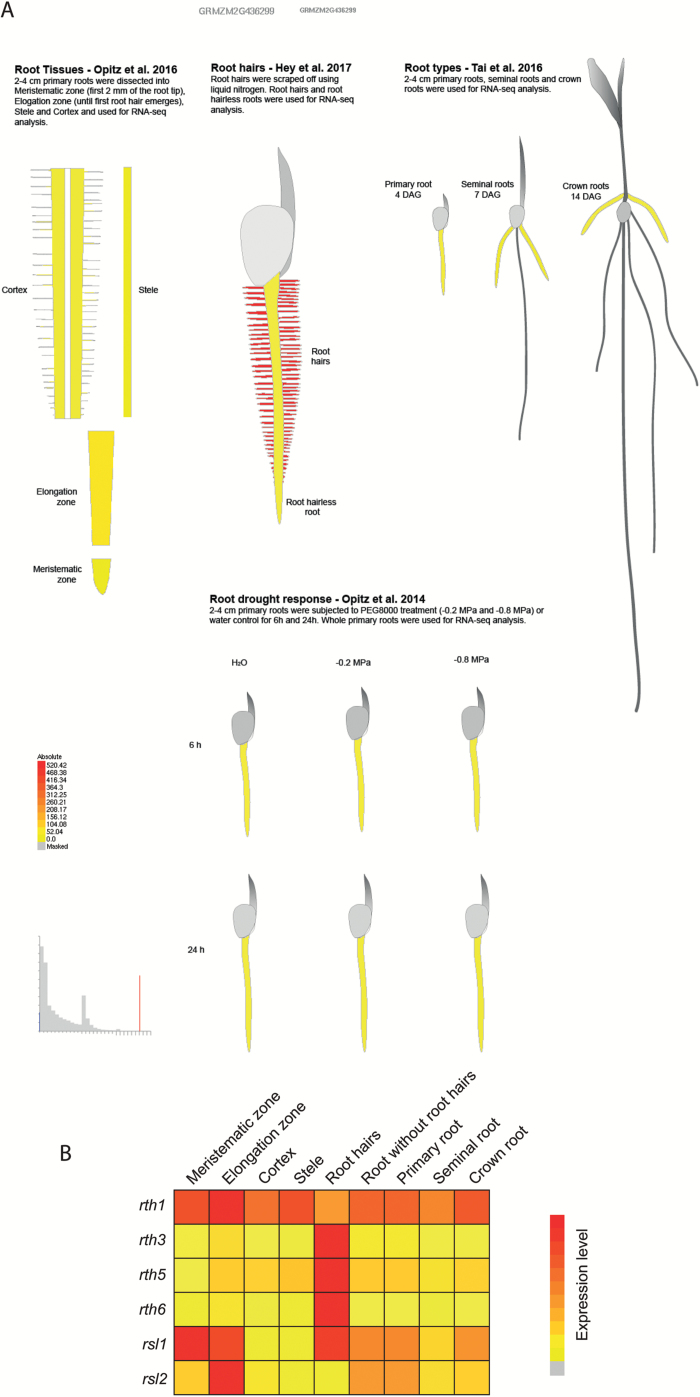
eFP browser. (A) New view of the eFP browser including the RNA-seq data set described in this study, RNA-seq data from different root tissues (Opitz *et al.*, 2016) and root-types (Tai *et al*., 2016). Furthermore, data of primary roots subjected to water deficit treatment were implemented (Opitz *et al.*, 2014). (B) Maize *rth1*, *rth3*, *rth5*, *rth6*, *rsl1*, and *rsl2* displayed in a compact view of the expression level extracted from the eFP browser.

To facilitate the comparability of the root hair transcriptome with gene expression in other tissues and organs, this data set was implemented into the maize eFP browser (Winter *et al.*, 2007) at http://bar.utoronto.ca/efp_maize/cgi-bin/efpWeb.cgi?dataSource=Maize_Root. The eFP browser provides color-coded expression levels in different tissues for any gene of interest. For a more comprehensive visualization of gene expression across the whole maize root system, we also uploaded other root-specific transcriptomic data sets generated in our laboratory. These included expression data of whole primary roots, seminal roots, and crown roots (Tai *et al.*, 2016), the root meristematic zone, elongation zone, cortex, and stele of primary roots (Opitz *et al.* 2016; control treatment), and whole primary roots subjected to mild (–0.2 MPa) and severe (–0.8 MPa) water deficit for 6 h and 24 h (Opitz *et al.*, 2014). As an example, the relative expression of *roothairless 6* (*rth6*) which has been recently demonstrated to be expressed specifically in root hairs (Li *et al.*, 2016) was visualized in the eFP browser (Fig. 3A). Expression values in this figure and subsequent heatmaps are displayed relative to its most prominent expression in a root type or tissue. Therefore, in this mode relative values can only be used to compare expression of a gene between different root types or tissues but not to compare expression between different genes. Data from the eFP browser on the other known maize genes involved in root hair elongation [*rth1* (Wen *et al.*, 2005), *rth3* (Hochholdinger *et al.*, 2008), and *rth5* (Nestler *et al.*, 2014)] have been summarized in a simplified relative heatmap (Fig. 3B). While *rth1* shows ubiquitous expression levels throughout the different root types and tissues, *rth3*, *rth5*, and *rth6* show root hair-specific expression. Moreover, the maize genes *rsl1* (AC216731.3_FG001) and *rsl2* (GRMZM2G066057) which are homologous to the Arabidopsis *RHD6* and *RSL1* genes have been included in the heatmap. These Arabidopsis genes and their homologs in the monocot species rice (Kim *et al.*, 2017) and *Brachypodium* (Kim and Dolan, 2016) function in root hair initiation. While maize *rsl1* (AC216731.3_FG001) shows the strongest expression in the meristematic zone, the elongation zone, and in root hairs, the expression of *rsl2* (GRMZM2G066057) peaks in the elongation zone and shows only very low expression in other tissues (Fig. 3B). These results illustrate that not all genes involved in root hair elongation are specifically expressed in root hairs. Together, the implementation of maize root transcriptome data in the eFP browser provides an easily accessible comprehensive resource for gene expression in maize roots.

### Phylogenetic relationship of maize and Arabidopsis root hair developmental genes

To date, no comprehensive list of maize genes homologous to Arabidopsis genes involved in root hair development is available. Therefore, we identified in the present study 170 maize homologs of 138 Arabidopsis root hair genes listed in the iRootHair database (Kwasniewski *et al.*, 2013) (Supplementary Table S4). Based on our differentially expressed gene analysis (FDR ≤1%), 59 of these genes were preferentially expressed in root hairs (positive values in Supplementary Table S4), while 65 of these genes were preferentially expressed in primary roots without root hairs (negative values in Supplementary Table S4). If no fold change is provided (Supplementary Table S4), the gene was either not differentially expressed or not expressed.

### Arabidopsis genes involved in differentiation of trichoblasts are not conserved in maize

The most obvious difference between Arabidopsis and maize root hair development is epidermal patterning. While patterning follows a strictly pre-determined position-dependent fate in Arabidopsis, patterning of root hairs in the maize epidermis is random. In Arabidopsis, the genes *SCM*, *WER*, *CPC*, and *GL2* are the most important regulators of epidermal cell differentiation. Remarkably, in the present study, maize homologs were only identified for the receptor-like kinase SCM (*ZmSCM1*, GRMZM2G434277; *ZmSCM2*, GRMZM2G335638; and *ZmSCM3*, GRMZM2G085246). All three homologs were preferentially expressed in non-root hair tissues (log_2_Fc –4.25, –3.14, and –2.73). Interestingly, no homologs were found for the other key genes involved in epidermal cell differentiation *WER*, *CPC*, and *GL2*. These observations could possibly indicate that epidermal patterning might follow different regulatory mechanisms in maize.

### Homologs of Arabidopsis genes involved in root hair initiation are present in the maize genome

**Fig. 4.**
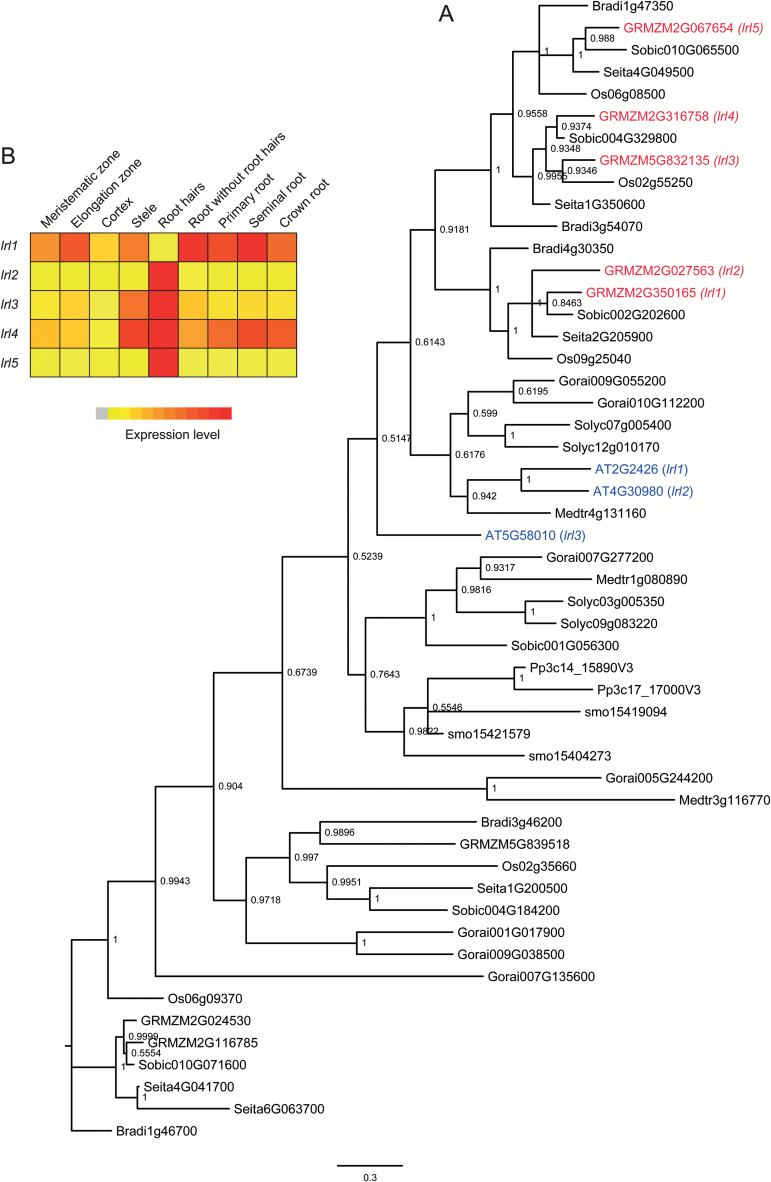
Phylogenetic tree of the Arabidopsis LRL genes. (A) Phylogenetic analysis of Arabidopsis LRL proteins. AtLRL1, AtLRL2, and AtLRL3 are highlighted in blue and maize homologs in red. The tree was constructed with full-length protein sequences using MrBayes. (B) Expression pattern of the five maize LRL genes extracted from the eFP browser.

**Fig. 5.**
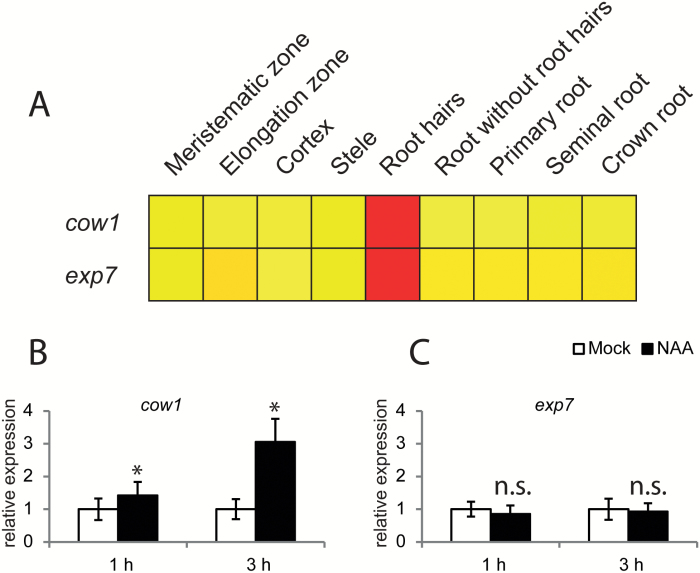
Auxin response and expression profiles of maize *cow1* and *exp7.* (A) *cow1* and *exp7* displayed in a compact view of the expression level extracted from the eFP browser. Expression levels of *cow1* (B) and *exp7* (C) were measured via qPCR in whole roots treated for 1 h or 3 h with auxin.

Root hair initiation is driven by a group of bHLH transcription factors. Phylogenetic analyses identified five maize genes homologous to the three Arabidopsis LRL bHLH transcription factors (*lrl1*, GRMZM2G350165; *lrl2*, GRMZM2G027563; *lrl3*, GRMZM5G832135; *lrl4*, GRMZM2G316758; and *lrl5*, GRMZM2G067654) (Fig. 4A). This adds one additional maize homolog to the previous study by Tam *et al.* (2015). Among those, four maize *lrl* genes were preferentially expressed in root hairs, while only *lrl1* was preferentially expressed in roots without root hairs (Fig. 4B; log_2_Fc –5.35; Supplementary Table S4). It has been previously demonstrated that Arabidopsis *LRL3* expression is auxin inducible, while Arabidopsis *LRL1* and *LRL2* do not respond to auxin treatment (Bruex *et al.*, 2012). To test auxin inducibility of the maize homologs, maize seedlings were treated with 5 µM 1-NAA for 1 h or 3 h. Gene expression was subsequently measured by qPCR. However, the expression of all genes was below the detection limit of qPCR. In Arabidopsis, auxin further induces *RSL2* and *RSL4* that in turn activate *COW1* and *EXP7* expression (Salazar-Henao *et al.*, 2016). Two maize homologs of *AtRSL2* and *AtRSL4* (designated *rsl3*, AC198518.3_FG005; and *rsl4*, GRMZM2G395549) were identified in this study. However, *rsl3* was not expressed in roots and *rsl4* was very weakly expressed and did not differ in expression in root hairs or primary roots without root hairs. Furthermore, two maize homologs of the Arabidopsis *COW1* gene were identified (*cow1*, GRMZM2G162461; and *cow2*, GRMZM2G171349), but only maize *cow1* expression was strongly enriched in root hairs (log_2_Fc=6) while *cow2* was expressed at very low levels in root hairs. For *AtEXP7*, one maize homolog (*exp7*, GRMZM2G127029) was identified which was preferentially expressed in root hairs (log_2_Fc=2.5) (Fig. 5A). To test if auxin induces the expression of the maize homologs *cow1* and *exp7*, the transcript levels were measured in auxin-treated seedling roots. Interestingly, only *cow1* was auxin inducible (Fig. 5B), while *exp7* did not exhibit increased expression (Fig. 5C). Together these data indicate that regulation and growth of root hairs in Arabidopsis and maize share common characteristics, but at the same time show differences during their development which need to be dissected in future genetic analyses.

## Discussion

### Root hairs: a model for single cell-type transcriptome studies in plants

Root hairs are tubular extensions of epidermis cell. Their simple accessibility allows them to be used as a model to study molecular processes on the single-cell level in plants. In contrast to root hairs representing a single cell type, primary roots with which root hairs are associated are made of multiple, functionally different cell types along the longitudinal and radial root axes (reviewed in Yu *et al.*, 2016*b*). Each cell type in composite roots displays a unique transcriptome profile (reviewed in Schnable *et al.*, 2004). Tissue-specific transcriptomic dynamics have been determined for maize root tissues (Paschold *et al.*, 2014; Baldauf *et al.*, 2016; Opitz *et al.*, 2016), on the level of single cell types in Arabidopsis (Birnbaum *et al.*, 2003; Brady *et al.*, 2007; Dinneny *et al.*, 2008), and individual root cell types of monocots such as pericycle cells of maize (Woll *et al.*, 2005; Dembinsky *et al.*, 2007; Yu *et al.*, 2016*a*). According to their low structural and functional complexity compared with whole roots, root hairs displayed low transcriptomic diversity. This is illustrated by fewer genes active or preferentially expressed in root hairs than in roots without root hairs (Fig. 1E, F). Only 3% of all genes expressed in this study were exclusively expressed in root hairs. This is similar to the numbers in Arabidopsis, where 4% of all genes were exclusively expressed in root hairs (Lan *et al.*, 2013). While in Arabidopsis, 27% of the genes were differentially expressed (FDR ≤5%; |log_2_Fc| ≥1) between root hairs and non-root hair samples (Li and Lan, 2015), 59% of all expressed genes were differentially expressed between root hairs and roots without root hairs in maize (FDR ≤1%; |log_2_Fc| ≥1) in the present study. These differences might be the consequence of different sampling procedures [root hair scraping in maize versus FACS (fluorescence-activated cell sorting) of protoplasts in Arabidopsis], numbers of biological replications and statistical procedures (linear mixed model from in maize versus read number cut-off in Arabidopsis)

### Functional annotation of the transcriptome illustrates high energy demand of root hairs

Root hairs are highly specialized cells, and as such show a functionally specialized transcriptome. Functional GO term annotation of transcriptomes allows a comprehensive view of prominent biological processes or pathways (Du *et al.*, 2010). While in Arabidopsis most root hair-specific genes were related to cell walls (Lan *et al.*, 2013), in maize no GO term related to cell walls was enriched. This might either be explained by the technical differences of the two analyses discussed above or may indicate that in maize cell wall biosynthesis is active in both root hair and primary root without root hair samples, while in Arabidopsis cell wall material was preferentially synthesized in root hairs but not in other tissues (Fig. 2). For the rapid tip growth of root hairs, energy availability is instrumental. These findings are in line with observations in Arabidopsis (Li and Lan, 2015). Furthermore, transcriptomic differences between Arabidopsis and maize root hairs could arise from the different sampling methods. While root hair breaking or scraping (in maize) collects only root hairs that are elongating and those that have reached their final size, FACS of green fluorescent protein (GFP)-tagged protoplasts (in Arabidopsis) also includes early trichoblasts.

Despite differences between the root hair transcriptomes of maize and Arabidopsis, they also displayed similarities. GO terms related to small GTPase-mediated signal transduction were enriched in maize root hairs. This pathway was also enriched in Arabidopsis (Lan *et al.*, 2013). Furthermore, in Arabidopsis, genes related to translation, nucleosome assembly, and response to cold were preferentially expressed in non-root hair tissue in root hairs (Lan *et al.*, 2013). Similar categories were also enriched in primary roots without root hairs in this study (Supplementary Fig. S1).

### Root hair-specific gene expression patterns

The genes *rth3*, *rth5*, and *rth6* display highly specific expression in root hairs (Fig. 3B). This is in line with the root hair-specific phenotype of these mutants (Hochholdinger *et al.*, 2008; Nestler *et al.*, 2014; Li *et al.*, 2016). In contrast, the *rth1* gene which also controls root hair elongation is expressed in all tissues surveyed thus far (Wen *et al.*, 2005). This is in line with the pleiotropic phenotype shown by the stunted plants of the *rth1* mutant (Wen and Schnable, 1994).

Genes involved in cell differentiation and root hair initiation might not be expressed in elongating root hairs such as those collected in this study (Wang *et al.*, 2016). In contrast, in Arabidopsis, root hair cells including early trichoblasts were collected for downstream analyses (Lan *et al.*, 2013). It has been reported that only a short expression peak of the Arabidopsis *RHD6* gene can be observed in the elongation zone, specifically in early trichoblasts (Menand *et al.*, 2007). Thus *RHD6* was preferentially expressed in root hair cells (Lan *et al.*, 2013). Double knockdown mutants of two of the three homologs of Arabidopsis, *RHD6* and *RSL1*, results in reduced root hair length in *Brachypodium* (Kim and Dolan, 2016) and rice (Kim *et al.*, 2017), suggesting partial functional redundancy. In fact, the expression of maize homologs of Arabidopsis *RHD6* and *RSL1* designated *rsl1* and *rsl2* peaks in the elongation zone (Fig. 3B), similar to observations in Arabidopsis, *Brachypodium*, and rice. These parallels indicate that these genes might also be involved in the root hair initiation, which needs to be substantiated by mutant analyses.

### Homologs of Arabidopsis genes in maize

During evolution, monocots and dicots were separated ~150 million years ago (Chaw *et al.*, 2004). While for many genes homologous genes are present in maize and Arabidopsis, there are also examples for genes involved in root hair development that are only present in one of the two species or for which no direct homologs can be assigned. For instance, the maize genes *rth3* (Hochholdinger *et al.*, 2008) and *rth5* (Nestler *et al.*, 2014) are located in monocot-specific clades and have no direct homologs in Arabidopsis. On the other hand, the maize gene *rth6* (Penning *et al.*, 2009) is a close homolog of the Arabidopsis genes CELLULOSE SYNTHASE LIKE D 3 (*csld3*) and CELLULOSE SYNTHASE LIKE D2 (*csld2*) (Favery *et al.*, 2001; Wang *et al.*, 2001) and the rice gene *csld1* (Kim *et al.*, 2007). In the same line of reasoning, other genes have been shown to fulfill homologous functions. The root hair-specific rice gene OsEXPA17 is a homolog of *AtEXP7*. These homologous genes are functionally redundant (Yu *et al.*, 2011).

It has been suggested that the number of *LRL* genes in Arabidopsis increased during evolution and that single copies have been dedicated to specific functions (Breuninger *et al.*, 2016). *AtLRL* genes show diverse expression patterns. None of them is specifically expressed in trichoblasts. However, all genes except *AtLRL4* were detected in root hairs (Breuninger *et al.*, 2016). Accordingly, double mutants of the *Physcomitrella* homologs *lrl1* and *lrl2* show defects in the development of the root hair-like tissues protonema and rhizoids, but their expression is not limited to these tissues (Tam *et al.*, 2015). In maize, only *lrl1* exhibited peak expression in the elongation zone, while *lrl2*, *lrl3*, *lrl4*, and *lrl5* were preferentially expressed in root hairs. In addition, maize *lrl3* and *lrl5* displayed considerable expression in stele tissue, suggesting that these genes might also be involved in other functions. Complementation studies or reverse genetic approaches will elucidate whether their expression peaks also relate to functions in root hair development.

### Role of auxin in root hair formation

Auxin plays a crucial role during root hair development (Velasquez *et al.*, 2016). The Arabidopsis *COW1* and *EXP7* genes are both auxin inducible, regulated by bHLH transcription factors (Bruex *et al.*, 2012). In contrast, in maize, only the *cow1* gene is auxin inducible, while *exp7* expression does not respond to auxin (Fig. 5). These genes are examples of differences for auxin-regulated gene expression between Arabidopsis and maize. Genetic analyses will provide further insights on the auxin regulatory pathway during root hair development.

In conclusion, identification of genes specifically and preferentially expressed in maize root hairs and their functional annotation extended our understanding of the development and functionality of root hairs. This study highlights the importance of energy metabolism in root hairs. Moreover, phylogenetic comparisons of homologous Arabidopsis and maize genes involved in root hair formation provide a starting point for future detailed genetic analysis of root hair formation in maize. Finally, the implementation of the root hair and other root transcriptome data sets into the eFP browser provides the maize community with a novel resource for a more in-depth analysis of gene function.

## Supplementary data

Supplementary data are available at *JXB* online.

Figure S1. GO term analysis of genes preferentially expressed in roots without root hairs.

Table S1. RNA-seq output and mapping results.

Table S2. List of expressed genes in root hairs (RH) and roots without root hairs (PR - RH).

Table S3. Full list of enriched GO terms of genes preferentially expressed in root hairs.

Table S4. Maize homologs for genes known to be involved in root hair development in Arabidopsis.

## Supplementary Material

supplementary_figure_S1

supplementary_table_S1

supplementary_table_S2

supplementary_table_S3

supplementary_table_S4
