# Supplementary material for: Complexity and specificity of the maize (Zea mays L.) root hair transcriptome
Source: J Exp Bot. 2017 Apr 8;68(9):2175–85. doi: 10.1093/jxb/erx104 (PMC5447894; doi:10.1093/jxb/erx104)
Supplement: supplementary_figure_S1 [file erx104_suppl_supplementary_figure_S1.pdf]

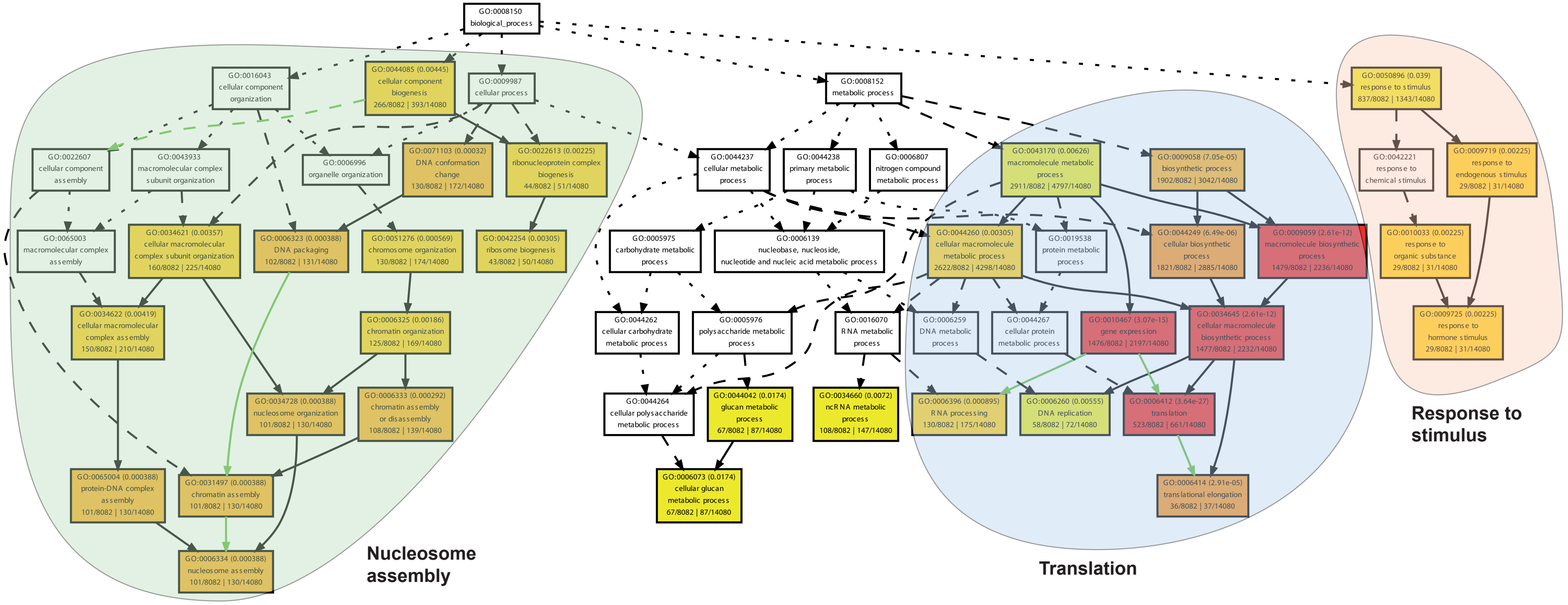

Supplementary figure S1. GO-term analysis of genes preferentially expressed in roots without root hairs. GO-term analysis of genes preferentially expressed in roots without root hairs. GO-terms related to one pathway are highlighted by colored clouds. Colors of individual boxes indicate significance level of enrichment. Significance levels are indicated in each box and range from yellow: FDR ≥ 0.05, dark red: p-value ≥ $1.8e^{-15}$.
